# Toxicity evaluation induced by single and 28-days repeated exposure of withametelin and daturaolone in Sprague Dawley rats

**DOI:** 10.3389/fphar.2022.999078

**Published:** 2022-09-26

**Authors:** Muhammad Waleed Baig, Muhammad Majid, Bakht Nasir, Syed Shams ul Hassan, Simona Bungau, Ihsan-ul Haq

**Affiliations:** ^1^ Department of Pharmacy, Quaid-i-Azam University, Islamabad, Pakistan; ^2^ Faculty of Pharmacy, Capital University of Science and Technology, Islamabad, Pakistan; ^3^ Charles Institute of Dermatology, School of Medicine, University College Dublin, Dublin, Ireland; ^4^ Shanghai Key Laboratory for Molecular Engineering of Chiral Drugs, School of Pharmacy, Shanghai Jiao Tong University, Shanghai, China; ^5^ Department of Natural Product Chemistry, School of Pharmacy, Shanghai Jiao Tong University, Shanghai, China; ^6^ Department of Pharmacy, Faculty of Medicine and Pharmacy, University of Oradea, Oradea, Romania

**Keywords:** withametelin, daturaolone, toxicity, *in vivo*, Datura

## Abstract

Safe preclinical dose determination is predictive of human toxicity and can have a profound impact on the overall progress of the compound in early drug discovery process. In this respect, current study sought to investigate for the first time the acute and subacute oral toxicity of two pharmacologically active natural compounds i.e., withametelin and daturaolone in Sprague Dawley rats following OECD guideline 420 and 407, respectively. As per acute toxicity studies, withametelin and daturaolone were characterized as Globally Harmonized System (GHS) category 4 and 5 compounds, respectively. Sub-acute daily dose of withametelin was 5, 2.5, and 1.25 mg/kg but, for daturaolone, it was 10, 5, and 2.5 mg/kg. High dose (5 and 2.5 mg/kg) withametelin groups showed dose dependent changes in the general, hematological, biochemical and histopathological parameters in both sexes, the most prominent being hyperthyroidism while no toxicity was observed at lower doses (1.25 and 0.75 mg/kg), No Observable Adverse Effect Level (NOAEL) being 1.25 mg/kg. Daturaolone was comparatively safer and showed dose dependent significant changes in hepatic enzyme (Alanine Transaminase), bilirubin, creatinine, and glucose levels while histological changes in testes were also observed. Lower doses (5, 2.5, and 1.25 mg/kg) of daturaolone showed no significant toxic effects and 5 mg/kg was declared as its NOAEL. Depending upon our findings, starting effective oral dose levels of 1.25 mg/kg/day for withametelin and 5 mg/kg/day for daturaolone are proposed for repeated dose (up to 28 days) preclinical pharmacological evaluation models. Long term studies with more behavioral, biochemical, histopathological and hormonal parameters are proposed to strengthen the findings.

## 1 Introduction

Natural products continue to contribute significantly to human health and longevity. They are promising pools for discovering high-strength structural diversity and different bioactive scaffolds have tendency to be developed directly or used as a starting point for optimizing new drugs (Khan et al., 2019; Hassan et al., 2021; Majid et al., 2022). One of the main criteria that applies to new drugs for research is to evaluate the safe dose and potential toxicity risk before human studies are carried out (Hassan et al., 2022; Shams ul Hassan et al., 2022). In this regard, pre-clinical studies estimate the initial dose of any drug candidate for advance testing (Shams ul Hassan et al., 2021). Withametelin (Baig et al., 2020) and daturaolone (Baig et al., 2021) were isolated in our previous studies. Withametelins is a subclass of withanolides mainly found in Datura genus (Chen et al., 2011; Muhammad et al., 2021). Withametelin possess antifungal (Singh et al., 2001) cytotoxicity against lung (Rao et al., 2016), liver, prostate and mammary cell lines (Baig et al., 2020). Its antileukemia effects are attributed to the inhibition of the spread of acute myeloid leukemia by targeting the signalling pathways of oncogenic kinase, which regulate the progression and survival of the cell cycle, thus resulting in an induction of apoptosis (Akhtar et al., 2020). It relieves vincristine-induced neuropathic pain by analyzing results from behavior, biochemistry, histopathology and computational data, the molecular mechanism being associated with the suppression of TRPV1/TRPM8/P2Y nociception and MAPK signalling in mice (Khan et al., 2021b). It also shows neuroprotective properties by modulating the oxidative stress by Nrf2/Keap1/HO-1 in the CNS and the neuroinflammatory stress by TLR4/IκB-α/NF-κB/AP-1 in the CNS (Khan et al., 2021a). Moreover, daturaolone is an amyrin type triterpenoid affective in fever, muscle relaxation and gastrointestinal problems (Bawazeer et al., 2020a). It significantly inhibits inflammation and cancer markers (NF-κB and NO, as shown in silico and *in vitro* models. In addition, powerful *in vivo* anti-inflammatory, anti-nociceptive and antidepressants had also been observed (Baig et al., 2021). Daturaolone also significantly inhibits α-glucosidase and β-secretase and can be used as an excellent template compound for new drugs used in diabetes and Alzheimer’s disease (Bawazeer et al., 2020b). Both natural compounds were proposed for their detailed mechanistic, toxicity profile, and clinical studies. Early detection of preclinical doses that predict human toxicity can have a significant impact on the development of compounds.

Financial investment increases exponentially as new chemicals move through discovery and development stages. Modern toxicologists can significantly influence expenditure by predicting safe doses and potential toxicological/side effects in advance (Lai, 2020; Obireddy and Lai, 2021a). Early detection of preclinical doses that are predictive of human toxicity can have a profound impact on the overall progression of the compound, its development costs, and clinical trials (Lai et al., 2021; Chen et al., 2022). Identifying preclinical and predictable safety liability at the beginning of the process might lead to better candidates being designed and selected (Anjum et al., 2017). Many efforts in toxicity research focus on the application of new technologies. Nevertheless, when separated from traditional (*in vivo*) toxicological principles, compounds cannot be developed. National and international regulators assess toxicological data to understand intrinsic toxicology of chemicals (Ji et al., 2020; Obireddy and Lai, 2021b). Effects of different doses in animal model systems is extrapolated to human exposure to understand potential risks. The main decisive factor is the determination of adverse reactions required to protect exposed people and the exposure levels at which these effects occur (Parasuraman, 2011).

In line with this, our study aimed to generate information on toxicity testing needed for the risk assessment of withametelin and daturaolone which has been identified in a study of oral toxicity in rodents by single dose and repeated dose for 28 days. A systematic toxicity assessment was carried out to generate data supporting the identification of appropriate starting doses and possible adverse reactions.

## 2 Material and methods

### 2.1 Animals

Sprague Dawley rats of either sex were procured from National Institute of Health (NIH). The overall wellbeing of rats was guaranteed. In the current study, one hundred forty two (142) rats aged seven to 8 weeks weighing in range of ∼190–220 g were used. Animals were placed in their respective aluminium cages with soft wood splinters on floor. Surroundings were maintained with temperature 23–25°C and air humidity of 50 ± 10% with a 12 h light/dark cycle. The bedding was regularly replaced with new wood splinters to avoid health risks and infections in research animals. Animals were housed in the Primate facility of Faculty of Biological Sciences, Quaid-i-Azam University Islamabad, Pakistan in compliance with the National Institute of Health, United States guidelines for the care and use of laboratory animals. They were provided with standard rodent food and water *ad libitum.* Study was conducted after ethical approval from the Institutional Animal Ethics Committee (letter number # BEC-FBS-QAU2019-135) and adhered with strict cautions to diminish animal distress.

### 2.2 Acute oral toxicity testing

Acute toxicity tests with only observational study were carried out for the selected doses of withametelin and daturaolone following the Organization for Economic Cooperation and Development (OECD) guideline 420, fixed-dose procedure with slight changes according to system suitability (Liu et al., 2016; Xiao et al., 2020). Principle is to use only moderately toxic doses and potentially fatal dose planning should be avoided. In addition, doses known to cause serious pain and suffering due to corrosion or serious irritation do not need to be administered. The initial doses chosen for observation studies were 5, 50, and 300 mg/kg. After a week of acclimatization, the animals were randomly divided into seven groups (3 males and three females). Group-I (vehicle control) was administered 10% DMSO in CMC. Group-II-IV were given different doses of withametelin while group-V-VII were administered selected doses of daturaolone. The rats were fasted overnight before dose and 2 h after treatment. The test material was administered by gavage in a single dose. The dose volume was calculated in advance based on the last body weight recorded for each animal. Toxicity signs such as change in body weight, ptosis, abnormal posture, abnormal respiration, diarrhoea and diuresis, lethargy, ataxia, abnormal gait, tremors, convulsion, prostrate, coma, lacrimation and exopthalmus were observed. The beginning and duration of observed toxic symptoms were systematically recorded. After dosing, the animal was monitored for 48 h to observe its survival and if any signs of toxicity occured. The first group of rats had no signs of toxicity and survived for 48 h. Therefore, rats in the second group received higher doses and were observed as before. The 300 mg/kg compound study took place because the second group of animals also survived more than 48 h. Three rats were administered with 300 mg/kg dose of withametelin and daturaolone and the survival time and toxic signs were observed. Animals surviving from highest dose were observed at least once for 14-days observational period. During this study, the body weight of the rats were measured and recorded on day 1, 7, and 14.

### 2.3 Repeated dose 28-day oral toxicity study

#### 2.3.1 Study design

**FIGURE 1:**
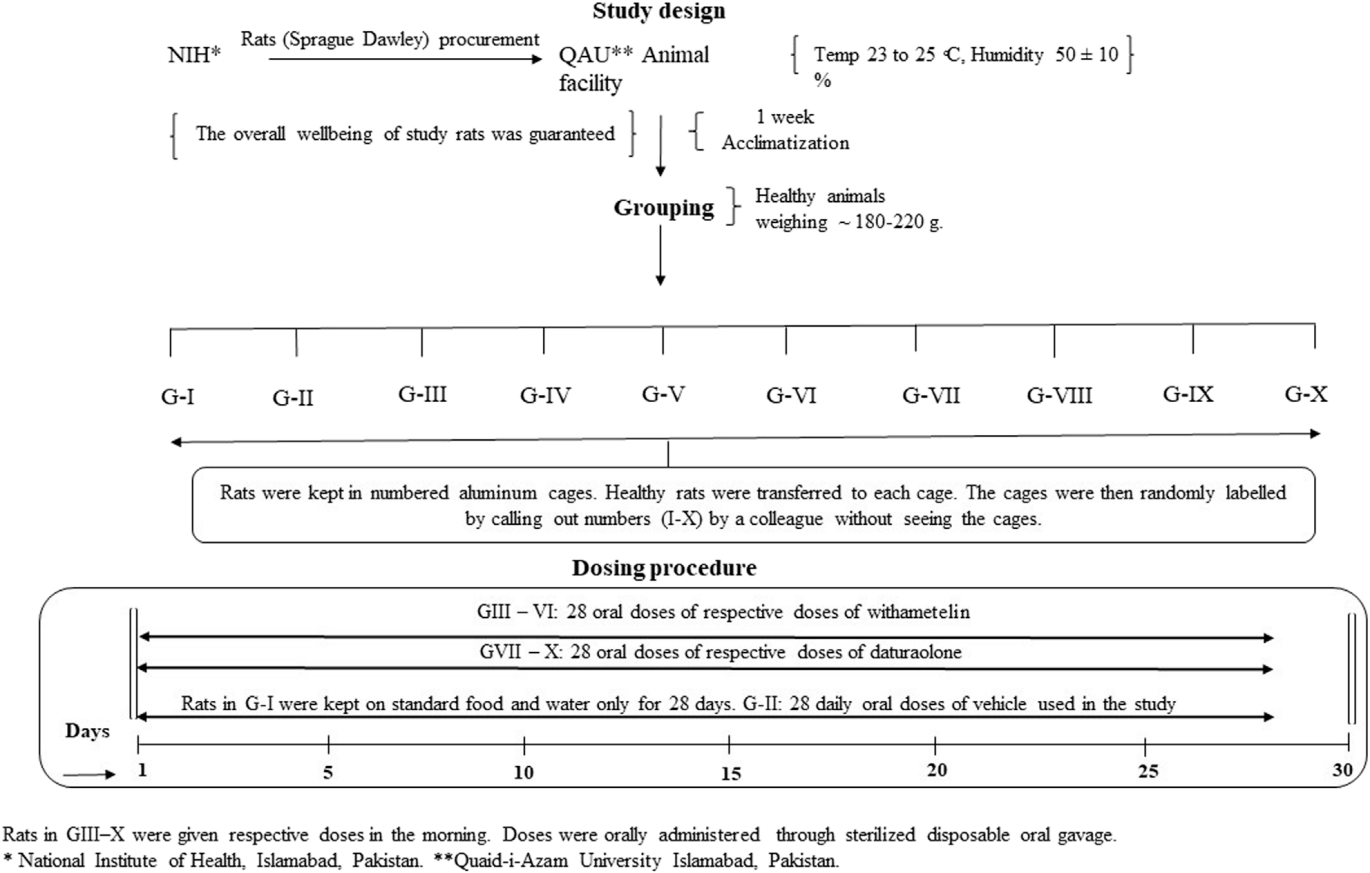
The experimental design of 28-days *in vivo* toxicity evaluation performed in Sprague Dawley rats.

A repeat dose toxicity study was conducted in rodents for selected doses of withametelin and daturaolone following the OECD guideline 407, Repeated Dose 28-Day Oral Toxicity procedure with slight modifications as per system suitability (Wu et al., 2015; Yimam et al., 2018) (Figure 1). After a week of acclimatization, rats were randomly divided into 10 groups, each group consisted of ten rats (5 males (M) and 5 females (F)). Rats were given oral gavage doses of withametelin (5, 2.5, 1.25, 0.75 mg/kg/day) and daturaolone (10, 5, 2.5, 1.25 mg/kg/day) at 7–8 weeks of age for at least 28 days. Doses were selected on the basis of our acute toxicity test and previously published *in vitro* and *in vivo* data. Negative control group was given only saline while vehicle control group was given only 10% DMSO in CMC. The daily dose volume was calculated in advance based on the most recent weight of each animal. The endpoints included clinical observations, body weight, water and feed consumption values, hematology, serum chemistry levels, thyroid levels and histopathological findings. Study design is as follows:

Group I: Normal control (Untreated, standard food only)
Group II: Vehicle control (10% DMSO in CMC)
Group III: Withametelin (5 mg/kg)
Group IV: Withametelin (2.5 mg/kg)
Group V: Withametelin (1.25 mg/kg)
Group VI: Withametelin (0.75 mg/kg)
Group VII: Daturaolone (10 mg/kg)
Group VIII: Daturaolone (5 mg/kg)
Group IX: Daturaolone (2.5 mg/kg)
Group X: Daturaolone (1.25 mg/kg)

#### 2.3.2 General observations

Clinical observations were made in all animals outside the house cage once before first administration of dose, thereafter, once every week and before necropsy. Signs sought included changes in skin, fur, eyes or mucous membranes, occurrence of secretions and excretions, lacrimation, piloerection, pupil size, respiratory pattern, changes in gait, posture, response to handling, excessive grooming and circling or bizarre behavior (self-mutilation, walking backwards).

#### 2.3.3 Body weight, food and water consumption

The weight of each rat was recorded once a week and immediately before the necropsy. Similarly, food and water consumption was measured weekly and results were expressed as mean ± SD.

#### 2.3.4 Collection of blood samples and serum separation

At the end of the experiment, rats were left without food for 24 h, breathed with isofluorane anesthesia, and were euthanized *via* cervical displacement. Blood samples were obtained from the heart of animals by intracardiac injection. Serum was separated from the collected blood samples by centrifugation at 4°C (6,000 rpm) for 15 min. The serum was stored at –20°C until biochemical testing was carried out.

#### 2.3.5 Estimation of hematological parameters

Blood samples were collected into sterile tubes with anticoagulant (EDTA-K_2_) for hematological tests. Hematological measurements include: red blood cells (RBC), hemoglobin (Hb), hematocrit (HCT), mean corpuscle volume (MCV), mean corpuscle hematocrit (MCHT), erythrocyte sedimentation rate (ESR), platelets, white blood cells (WBC), lymphocyte, monocyte, neutrophil, eosinophil using automatic hematology analyzer (Díez-Quijada et al., 2021).

#### 2.3.6 Assessment of biochemical parameters

Blood samples were collected into sterile tubes without anticoagulant for biochemical tests (serum). Alanine transaminase (ALT), aspartate aminotransferase (AST), alkaline phosphatase (ALP), bilirubin, creatinine, albumin, total serum protein (protein), urea, cholesterol and triglycerides using automatic chemistry analyzer (Roche Diagnostics, IN, United States) (Nasir et al., 2020; Díez-Quijada et al., 2021).

#### 2.3.7 Hormonal analysis

Triiodothyronine (T3), thyroxine (T4), and thyroid-stimulating hormone (TSH) were measured at the end of the test period. T3 and T4 were determined using the automatic immunoassay system (IMX, Abbott Laboratories, Abbott Park, Ill., United States), and TSH was measured with a microplate reader (UVmax, Molecular Devices, Sunnyvale, Calif., United States) (Díez-Quijada et al., 2021).

#### 2.3.8 Organ weights

The following organs were weighed at necropsy: brain, kidney, heart, liver, lungs, spleen, testis, ovary and uterus**.**

#### 2.3.9 Histological investigation (hematoxylin-eosin staining)

The following organs were fixed in 10% neutral buffered formalin and examined: heart, liver, kidney, testes and intestine. Evaluation of any change in histology was carried out using paraffin-associated staining protocols. After the dissection, the tissues were fixed for 12 h at room temperature with a buffer solution of 10% formalin (pH 7.4). The removal of the infiltrating wax and water traces was guaranteed via numerous ethanol (50, 70, 90, and 100%) washings of the fixed tissue. Small pieces of the incorporated tissue (3–5 m thick) were divided to prepare slides and latter stained with Eosin and Hematoxylin (H and E) (Majid et al., 2019).

### 2.4 Statistical analysis

The data were analyzed using one-way variation analysis (ANOVA) and then subsequently appropriate post-hoc tests. The results are represented as the mean ± SD of the respective parameters and *p* < 0.05 was considered statistically significant.

## 3 Results

### 3.1 Acute toxicity assessment

No changes in clinical symptoms, such as external appearance, behaviour, mental state and daily activities, were observed due to treatment except at 300 mg/kg withametelin group (Table 1). Morbidity was observed in 2/3 animals up to day 5 but no animal died even at a dose of 300 mg/kg (Table 2). Change in weight, abnormal posture, abnormal gait and diarrhoea were observed in 300 mg/kg group. Changes in weight clearly showed some damage caused by test material. Withametelin was characterized as GHS (globally harmonized classification system in mg/kg body weight) category 4 compound with evident toxicity at 300 mg/kg in acute toxicity assessment. Observable parameters and weight changes in different dose groups are mentioned in Table 1. In Daturaolone, no changes related to treatment have been observed (Table 1). A soft fecal consistency was observed in all rats in the treatment groups; however, this symptom was not observed on 2nd day after administration. No changes were observed after gross necroscopy of both compounds (data not shown). As per observations, it was assumed that daturaolone was GHS category 5 or unclassified compound which might show toxicity at 2000 mg/kg. Experiment was not performed at 2000 mg/kg due to insufficient quantity of compound and requirement to observe the effects of multiple daily dosing of daturaolone on rats.

**TABLE 1:** Acute toxicity assessment of withametelin and daturaolone at different doses.

Parameters	Days (1-14)						
	Withametelin					Daturaolone	
	Vehicle control	5 mg/kg	50 mg/kg	300 mg/kg	5 mg/kg	50 mg/kg	300 mg/kg
Δ Body weight	✗	✗	✗	**✓**	✗	✗	✗
Ptosis	✗	✗	✗	✗	✗	✗	✗
Abnormal Posture	✗	✗	✗	**✓**	✗	✗	✗
Abnormal respiration	✗	✗	✗	✗	✗	✗	✗
Diarrhoea and diuresis	✗	✗	✗	**✓**	✗	✗	✗
Lethargy	✗	✗	✗	**✓**	✗	✗	✗
Ataxia	✗	✗	✗	✗	✗	✗	✗
Abnormal gait	✗	✗	✗	**✓**	✗	✗	✗
Tremors	✗	✗	✗	✗	✗	✗	✗
Convulsion	✗	✗	✗	✗	✗	✗	✗
Prostrate Coma	✗	✗	✗	✗	✗	✗	✗
Lacrimation	✗	✗	✗	✗	✗	✗	✗
Exopthalmus	✗	✗	✗	✗	✗	✗	✗

N = 4. ✗ = absent, **✓** = present.

**TABLE 2:** Body weight, morbidity, and mortality after acute toxicity studies of withametelin and daturaolone at selected doses.

	Withametelin (mg/kg)				Daturaolone (mg/kg)		
	0	5	50	300	5	50	300
Body weight (g)							
Day 1	210.8 ± 4.24	215.36 ± 4.32	214.21 ± 3.23	215.63 ± 3.96	218.29 ± 2.34	215.67 ± 5.61	217.89 ± 5.58
Day 7	232.4 ± 3.76	236.38 ± 5.48	239.21 ± 3.76	225 ± 4.32	239.23 ± 4.45	235.14 ± 3.39	237.85 ± 5.11
Day 14	256.4 ± 3.76	261.31 ± 5.48	252.40 ± 5.31	238 ± 5.17	264.46 ± 3.90	261.29 ± 4.22	258.39 ± 6.16
Morbidity							
Day 1	0/3	0/3	0/3	0/3	0/3	0/3	0/3
Day 7	0/3	0/3	0/3	2/3	0/3	0/3	0/3
Day 14	0/3	0/3	0/3	0/3	0/3	0/3	0/3
Mortality							
Day 1	0/3	0/3	0/3	0/3	0/3	0/3	0/3
Day 7	0/3	0/3	0/3	0/3	0/3	0/3	0/3
Day 14	0/3	0/3	0/3	0/3	0/3	0/3	0/3

### 3.2 Repeated dose sub-acute toxicity *in vivo*

#### 3.2.1 General observations

No death was seen in all males and females of withametelin and daturaolone groups. The increased incidence of post-dosing salivation was observed in all treatment groups of withametelin. The softness in fecal consistency was observed on fifth day in all groups (withametelin) which gradually eased over 2 weeks. The control group had neither diarrhea nor melena. Abnormal gait (2/5 males) in last week was also seen with piloerection. In case of daturaolone, no general observational symptom was observed at all doses.

#### 3.2.2 Body weight, food, and water consumption

**FIGURE 2:**
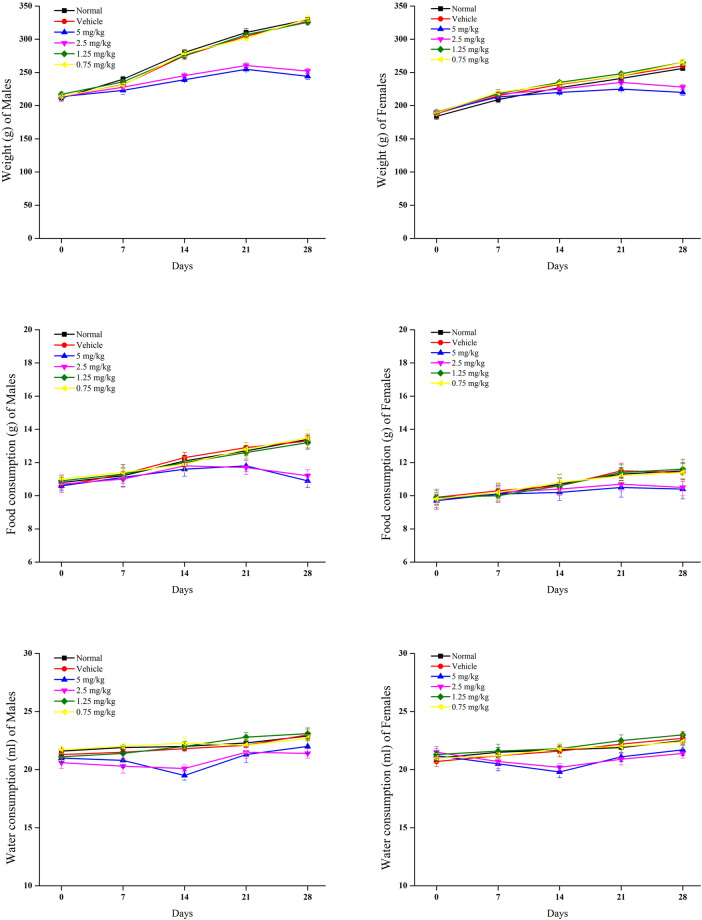
Mean food intake, water consumption, and weekly weight gain of rats in 28 days feeding study of withametelin.

**FIGURE 3:**
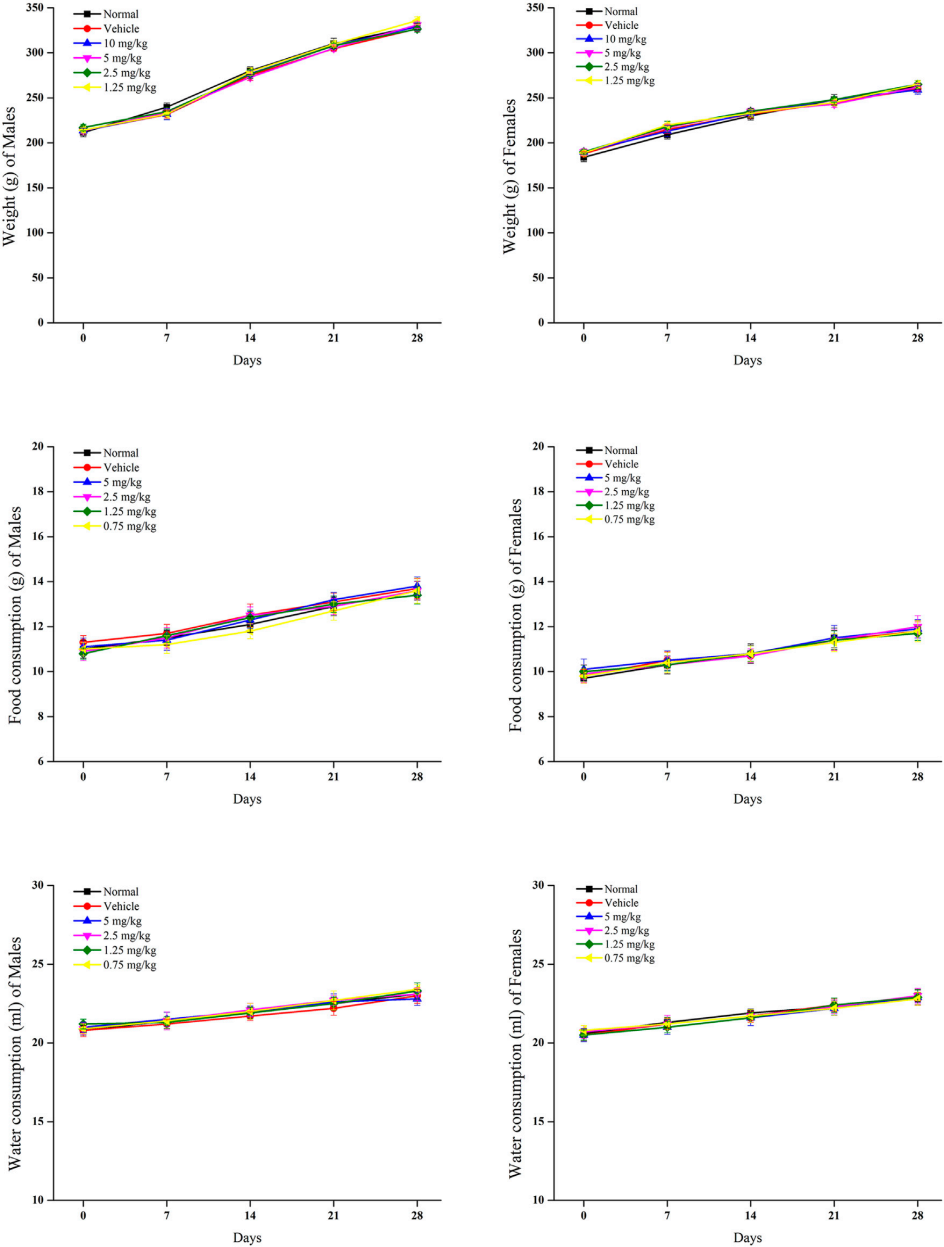
Mean food intake, water consumption, and weekly weight gain of rats in 28 days feeding study of daturaolone.

In relation to the control group, rat body weights were significantly reduced in groups of 5 and 2.5 mg/kg withametelin (Figure 2). A significant weight reduction was observed in males between 2 and 4 weeks, respectively while a significant reduction in body weight was observed in the female group during the 3rd to 4th week, respectively. In the 1.25 mg/kg and 0.75 mg/kg withametelin groups, the weight difference was not significant compared to the control group. (Figure 2A). At 5 and 2.5 mg/kg withametelin groups, and at 3 and 4 weeks, the consumption of the food of male and female rats was significantly reduced. Compared to control rats, there was no significant decrease in food efficiency between female and male rats who were fed 1.25 mg/kg and 0.75 mg/kg withametelin (Figure 2B). Water consumption decreased considerably when noted on 14th day, but then it became normal in the third and fourth week (Figure 2). Significant weight reduction observed in high-dosed male rats was due to the administration of 5 mg and 2.5 mg/kg of withametelin. Since water consumption changes were temporary and had low magnitude, they were not considered to be associated with the treatment. In the case of daturaolone, male and female rats groups of all doses showed no significant changes in weight and nutrition parameters (Figure 3).

#### 3.2.3 Hematological paramaeters

Hematological analysis results are presented in Table 3. We observed significant changes in RBC associated parameters, while a significant increase of platelets and neutrophils in withametelin (5 mg/kg) and daturaolone (10 mg/kg) treated rats. The level of platelets remained high at 2.5 mg/kg withametelin, but lower than the 5 mg/kg-withametelin group. The hematology indices values in high-dose groups with withametelin and daturaolone that do not correspond to the normal control have dose dependent clinical significance or toxicity. There was no significant difference between WBCs and its associated response variables or red cell indices between treated and non-treated rats at lower doses of both compounds, respectively.

**TABLE 3:** Hematology results after 28 days respectively.

Parameter	Sex	Hematological data									
		Withametelin						Daturaolone			
		Normal	Vehicle	5	2.5	1.25	0.75	10	5	2.5	1.25
RBC (×10^6^)/µL	M	6.91 ± 0.98	6.74 ± 0.96	6.14 ± 0.91*	6.87 ± 0.57	7.56 ± 0.76	6.98 ± 0.78	6.21 ± 0.56	6.83 ± 0.78	6.98 ± 0.75	6.65 ± 0.82
	F	7.73 ± 1.21	7.35 ± 1.67	6.26 ± 1.21*	7.03 ± 1.45	7.78 ± 2.49	7.64 ± 1.69	7.19 ± 1.65	7.60 ± 1.63	7.77 ± 2.64	7.68 ± 1.64
Hb (g/dl)	M	14.5 ± 1.23	14.77 ± 2.14	11.53 ± 1.23*	13.93 ± 1.38	14.83 ± 1.63	13.89 ± 1.64	12.33 ± 1.34*	13.96 ± 2.14	14.32 ± 1.63	13.81 ± 1.78
	F	16.1 ± 1.25	16.86 ± 2.13	16.7 ± 1.27*	15.8 ± 1.44	15.94 ± 1.35	16.78 ± 1.65	14.90 ± 1.45*	16.92 ± 2.45	16.71 ± 1.66	16.74 ± 1.56
HCT (%)	M	48.8 ± 2.32	49.82 ± 4.33	42.3 ± 2.47*	46.4 ± 2.43	50.76 ± 3.76	49.09 ± 2.79	40.10 ± 2.78*	50.23 ± 4.76	50.74 ± 3.78	49.02 ± 2.36
	F	52.0 ± 3.08	53.08 ± 3.45	45.5 ± 2.76*	51.6 ± 3.00	53.02 ± 4.73	52.04 ± 8.72	45.39 ± 3.98*	57.52 ± 3.23	53.12 ± 4.72	52.05 ± 8.74
MCV (fL)	M	69.9 ± 3.32	71.85 ± 3.37	60.32 ± 4.12*	68.40 ± 5.37	71.95 ± 4.78	68.98 ± 2.88	72.67 ± 3.26	72.20 ± 3.54	71.45 ± 4.79	68.91 ± 2.63
	F	66.73 ± 2.98	68.53 ± 3.98	57.35 ± 2.76*	63.77 ± 3.65	66.73 ± 2.98	68.98 ± 2.18	70.20 ± 2.97	68.94 ± 3.26	66.54 ± 2.91	68.99 ± 2.26
MCHC (%)	M	30.04 ± 1.20	29.67 ± 2.22	32.32 ± 1.45	30.5 ± 2.76	31.39 ± 2.68	31.34 ± 4.76	26.45 ± 1.15	28.39 ± 2.15	31.24 ± 2.64	31.35 ± 4.64
	F	31.43 ± 2.23	32.12 ± 1.93	28.43 ± 2.65*	30.34 ± 3.19	33.73 ± 2.45	34.09 ± 1.76	27.87 ± 2.46	32.65 ± 1.35	33.43 ± 2.49	34.01 ± 1.57
ESR (mm/hr)	M	3.98 ± 0.08	3.76 ± 0.05	4.19 ± 0.09	3.80 ± 0.07	3.96 ± 0.03	3.87 ± 0.04	3.78 ± 0.08	3.82 ± 0.03	3.92 ± 0.01	3.82 ± 0.03
	F	4.23 ± 0.06	4.10 ± 0.09	4.33 ± 0.07	4.25 ± 0.05	4.09 ± 0.05	3.95 ± 0.03	3.74 ± 0.05	3.98 ± 0.06	3.78 ± 0.04	3.93 ± 0.02
Platelets (×10^3^)/µL	M	1358 ± 176	1420 ± 189	1892 ± 151*	1677 ± 223*	1315 ± 279	1402 ± 254	1665 ± 157*	1410 ± 163	1480 ± 270	1434 ± 246
	F	1262 ± 152	1356 ± 174	1774 ± 170*	1554 ± 235*	1369 ± 234	1323 ± 245	1590 ± 162*	1309 ± 153	1354 ± 232	1325 ± 227
WBC (×10^3^)/µL	M	9.42 ± 1.34	10.32 ± 1.51	12.09 ± 1.51*	11.49 ± 2.76*	9.54 ± 1.74	9.74 ± 1.87	12.97 ± 1.54*	9.89 ± 1.47	9.51 ± 1.78	9.79 ± 1.74
	F	7.23 ± 0.87	8.56 ± 1.85	10.29 ± 0.70*	9.94 ± 0.87*	7.49 ± 0.90	7.41 ± 0.67	9.36 ± 0.86*	8.19 ± 1.68	7.47 ± 0.98	7.49 ± 0.63
Lymphocyte %	M	80.25 ± 4.21	83.25 ± 3.29	85.63 ± 5.36	82.44 ± 3.76	82.24 ± 4.29	80.29 ± 3.10	80.44 ± 4.26	81.47 ± 3.11	82.28 ± 4.21	80.23 ± 3.15
	F	82.16 ± 3.43	85.14 ± 4.67	88.82 ± 5.49	83.12 ± 3.56	84.15 ± 3.76	82.17 ± 3.44	81.98 ± 3.87	83.39 ± 4.78	84.35 ± 3.73	82.15 ± 3.56
Monocyte	M	0.20 ± 0.02	0.22 ± 0.06	0.21 ± 0.02	0.19 ± 0.03	0.18 ± 0.01	0.20 ± 0.01	0.23 ± 0.02	0.22 ± 0.08	0.19 ± 0.02	0.21 ± 0.02
	F	0.18 ± 0.01	0.19 ± 0.03	0.18 ± 0.01	0.16 ± 0.04	0.15 ± 0.07	0.18 ± 0.06	0.20 ± 0.01	0.20 ± 0.04	0.18 ± 0.05	0.17 ± 0.04
Neutrophil %	M	11.3 ± 1.43	10.5 ± 1.65	19.6 ± 2.87*	15.21 ± 1.56*	10.9 ± 2.22	11.4 ± 2.98	14.78 ± 1.67*	10.78 ± 1.58	9.59 ± 2.28	11.24 ± 2.78
	F	8.29 ± 2.30	7.97 ± 1.69	17.2 ± 1.36*	12.96 ± 3.44*	8.46 ± 1.35	8.04 ± 1.43	11.19 ± 2.55*	8.54 ± 2.55	7.88 ± 1.31	7.88 ± 1.31
	F	1.27 ± 0.05	1.64 ± 0.09	1.44 ± 0.06	1.25 ± 0.08	1.16 ± 0.02	1.26 ± 0.03	1.32 ± 0.02	1.47 ± 0.07	1.16 ± 0.01	1.21 ± 0.04
Eosinophil %	M	2.21 ± 0.21	2.34 ± 0.29	2.03 ± 0.34	2.34 ± 0.34	2.34 ± 0.26	2.29 ± 0.54	2.27 ± 0.17	2.30 ± 0.21	2.29 ± 0.29	2.27 ± 0.49
	F	1.98 ± 0.68	2.10 ± 0.77	1.76 ± 0.54	1.99 ± 0.67	1.99 ± 0.62	1.84 ± 0.59	2.07 ± 0.49	1.88 ± 0.63	1.97 ± 0.54	1.80 ± 0.43

Results are expressed as mean ± SD.

*p < 0.05 was considered significantly different as compared with the controls.

#### 3.2.4 Biochemical parameters

Biochemical analysis result values are presented in Table 4. In withametelin dosed 5 and 2.5 mg/kg groups, significant increase of ALT and AST were observed while plasma glucose levels were significantly low in comparison to control. In thyroid profile, low TSH and high T3 and T4 was observed. Cholesterol and triglyceride levels were also lowered in 5 mg/kg withametelin groups. All other tested biochemical parameters were normal. In daturaolone dosed 10 mg/kg male and female groups, significant difference was noted regarding ALT, bilirubin, creatinine and glucose indices between treated and untreated rats. Insignificant changes in thyroid profile i.e., low TSH and high T3 and T4 were observed Biochemical parameter values in high dose withametelin and daturaolone groups that are not within the normal range have shown dose dependent clinical significance or toxicity.

**TABLE 4:** Serum clinical chemistry of rats sacrificed after 28-days repeated oral dose toxicity evaluation.

Parameter	Sex	Biochemical Data									
		Withametelin						Daturaolone			
		Normal	Vehicle	5	2.5	1.25	0.75	10	5	2.5	1.25
ALT U/L	M	29.31 ± 3.42	28.72 ± 2.91	39.91 ± 3.41*	35.14 ± 3.4*	30.36 ± 3.21	31.26 ± 2.56	34.89 ± 3.45*	30.14 ± 2.94	30.43 ± 3.23	29.30 ± 2.59
	F	22.52 ± 3.93	21.23 ± 2.18	32.26 ± 2.54*	29.87 ± 3.9*	31.51 ± 4.56	28.20 ± 4.21	27.97 ± 5.23*	25.67 ± 3.36	31.53 ± 4.65	28.65 ± 4.22
AST U/L	M	123.43 ± 3.98	133.52 ± 5.23	143.07 ± 4.21*	147.92 ± 7.24*	120.20 ± 4.67	125.87 ± 4.87	132.13 ± 5.67	125.87 ± 5.21	120.53 ± 4.83	125.54 ± 4.85
	F	110.97 ± 5.21	121.43 ± 6.42	135.45 ± 3.22*	139.53 ± 6.35*	107.57 ± 6.21	114.65 ± 4.86	120.78 ± 8.96	127.72 ± 5.55	107.63 ± 6.81	114.23 ± 4.82
ALP U/L	M	226.08 ± 10.23	237.98 ± 15.23	201.57 ± 15.68*	190.86 ± 13.74*	220.10 ± 12.46	222.71 ± 12.30	238.76 ± 13.38	240.92 ± 10.30	220.67 ± 12.93	222.62 ± 12.35
	F	150.97 ± 15.21	166.82 ± 8.77	133.93 ± 19.41*	141.83 ± 10.83*	155.71 ± 16.82	154.87 ± 16.71	170.87 ± 14.30	151.87 ± 6.21	155.87 ± 16.57	154.12 ± 16.76
Bilirubin mg/mL	M	3.82 ± 0.76	3.23 ± 1.23	3.56 ± 0.81	3.54 ± 0.79	3.15 ± 0.53	3.17 ± 0.62	4.80 ± 0.53*	3.68 ± 0.43	3.27 ± 0.47	3.45 ± 0.60
	F	2.43 ± 0.58	2.74 ± 0.93	2.63 ± 1.12	2.78 ± 0.91	2.56 ± 0.71	2.64 ± 0.54	3.58 ± 0.43*	2.73 ± 0.26	2.59 ± 0.54	2.34 ± 0.51
Albumin mg/dL	M	3.96 ± 0.94	3.87 ± 0.76	3.90 ± 0.78	3.99 ± 0.53	3.78 ± 0.48	3.99 ± 0.70	3.88 ± 0.62	3.68 ± 0.76	3.82 ± 0.51	3.67 ± 0.72
	F	4.17 ± 0.68	4.23 ± 0.54	3.61 ± 0.51	3.92 ± 0.78	3.80 ± 0.62	4.23 ± 0.39	4.37 ± 0.63	4.14 ± 0.52	3.78 ± 0.59	4.74 ± 0.33
Urea mg/dL	M	29.23 ± 1.78	31.70 ± 2.79	28.46 ± 1.48	39.87 ± 2.30	28.44 ± 3.34	30.44 ± 3.22	30.65 ± 3.67	29.89 ± 2.87	28.48 ± 3.40	30.83 ± 3.25
	F	30.26 ± 2.84	29.74 ± 4.32	30.78 ± 5.75	28.03 ± 3.34	29.30 ± 4.43	29.73 ± 3.21	32.11 ± 1.47	32.76 ± 3.62	29.28 ± 4.36	29.82 ± 3.22
BUN mg/dL	M	14.44 ± 1.19	15.23 ± 2.39	14.22 ± 2.19	15.25 ± 2.49	14.11 ± 1.18	15.23 ± 2.14	15.87 ± 2.30	16.12 ± 1.53	14.17 ± 1.21	15.54 ± 2.15
	F	15.17 ± 1.20	14.28 ± 2.30	13.47 ± 2.34	14.92 ± 1.37	14.25 ± 2.34	15.45 ± 3.33	16.24 ± 2.94	14.78 ± 1.67	14.32 ± 2.41	15.25 ± 3.32
Creatinine mg/dL	M	0.83 ± 0.13	0.89 ± 0.16	0.61 ± 0.57*	0.87 ± 0.19	0.82 ± 0.10	0.80 ± 0.14	1.93 ± 0.21*	0.90 ± 0.31	0.85 ± 0.09	0.57 ± 0.16
	F	0.81 ± 0.24	0.86 ± 0.27	0.57 ± 0.34*	0.86 ± 0.27	0.80 ± 0.16	0.79 ± 0.14	1.75 ± 0.30*	0.88 ± 0.07	0.82 ± 0.15	0.28 ± 0.17
Protein mg/dL	M	6.97 ± 0.51	6.74 ± 0.54	6.99 ± 0.76	6.12 ± 0.64	7.19 ± 0.76	6.95 ± 0.75	6.72 ± 0.87	6.75 ± 0.32	7.24 ± 0.71	6.34 ± 0.71
	F	6.73 ± 0.52	6.64 ± 0.49	6.34 ± 0.54	6.42 ± 0.46	6.89 ± 0.43	6.72 ± 0.86	6.79 ± 0.25	6.82 ± 0.24	6.94 ± 0.45	6.83 ± 0.83
Glucose mg/dL	M	82.78 ± 10.25	85.34 ± 9.54	68.49 ± 8.27*	73.29 ± 5.49*	86.19 ± 8.36	84.29 ± 11.49	65.93 ± 6.92*	81.78 ± 9.63	86.54 ± 8.46	84.25 ± 11.42
	F	94.56 ± 12.68	99.46 ± 10.89	75.24 ± 8.47*	83.72 ± 6.37*	91.24 ± 11.24	96.58 ± 9.34	78.48 ± 9.65*	92.54 ± 9.42	91.38 ± 11.12	96.63 ± 9.36
Cholesterol mMol/L	M	74.90 ± 4.91	77.64 ± 2.49	68.47 ± 2.31*	71.88 ± 3.43*	73.79 ± 6.24	75.55 ± 4.17	76.81 ± 4.86	80.32 ± 3.49	73.82 ± 6.66	75.28 ± 4.13
	F	55.81 ± 3.67	59.76 ± 4.59	49.18 ± 2.72*	51.37 ± 2.99*	57.86 ± 3.08	58.79 ± 3.52	63.44 ± 3.84	61.78 ± 4.38	57.89 ± 3.11	58.72 ± 3.55
Triglycerides mMol/L	M	90.29 ± 4.29	92.36 ± 5.19	83.46 ± 5.16*	81.79 ± 3.47*	89.24 ± 3.57	91.81 ± 6.76	96.19 ± 4.98	92.41 ± 3.81	89.62 ± 3.63	91.61 ± 6.71
	F	72.39 ± 5.18	77.31 ± 4.48	65.48 ± 7.64*	68.59 ± 6.64*	75.91 ± 6.49	74.22 ± 4.39	77.90 ± 5.92	74.21 ± 5.84	75.84 ± 6.50	74.48 ± 4.38
TSH ng/mL	M	4.82 ± 0.24	4.79 ± 0.29	3.14 ± 0.11*	3.88 ± 0.71*	4.70 ± 0.51	4.84 ± 0.43	4.58 ± 0.46	4.93 ± 0.25	4.69 ± 0.52	4.37 ± 0.41
	F	4.61 ± 0.34	4.66 ± 0.75	3.39 ± 0.26*	4.09 ± 0.64*	4.58 ± 0.25	4.65 ± 0.53	4.42 ± 0.37	4.54 ± 0.34	4.63 ± 0.26	4.52 ± 0.56
T3 ng/mL	M	1.75 ± 0.09	1.79 ± 0.24	1.88 ± 0.43*	1.80 ± 0.72*	1.73 ± 0.41	1.76 ± 0.53	1.73 ± 0.04	1.55 ± 0.28	1.69 ± 0.45	1.68 ± 0.51
	F	1.69 ± 0.10	1.74 ± 0.35	1.79 ± 0.58*	1.77 ± 0.57*	1.70 ± 0.23	1.61 ± 0.47	1.80 ± 0.16	1.59 ± 0.30	1.60 ± 0.26	1.63 ± 0.48
T4 ng/mL	M	60.47 ± 3.47	61.30 ± 4.76	81.24 ± 4.39*	75.73 ± 6.97*	64.19 ± 2.16	62.29 ± 4.13	65.53 ± 2.88	57.40 ± 3.76	57.38 ± 2.14	62.31 ± 4.12
	F	58.76 ± 2.64	63.67 ± 4.87	78.61 ± 5.37*	73.49 ± 6.84*	65.29 ± 3.42	60.94 ± 4.62	67.87 ± 3.52	54.63 ± 6.23	60.73 ± 3.49	60.48 ± 4.67

Results are expressed as mean ± SD.

*p < 0.05 was considered significantly different as compared with the controls.

#### 3.2.5 Relative organ weight and histopathological investigation

**FIGURE 4:**
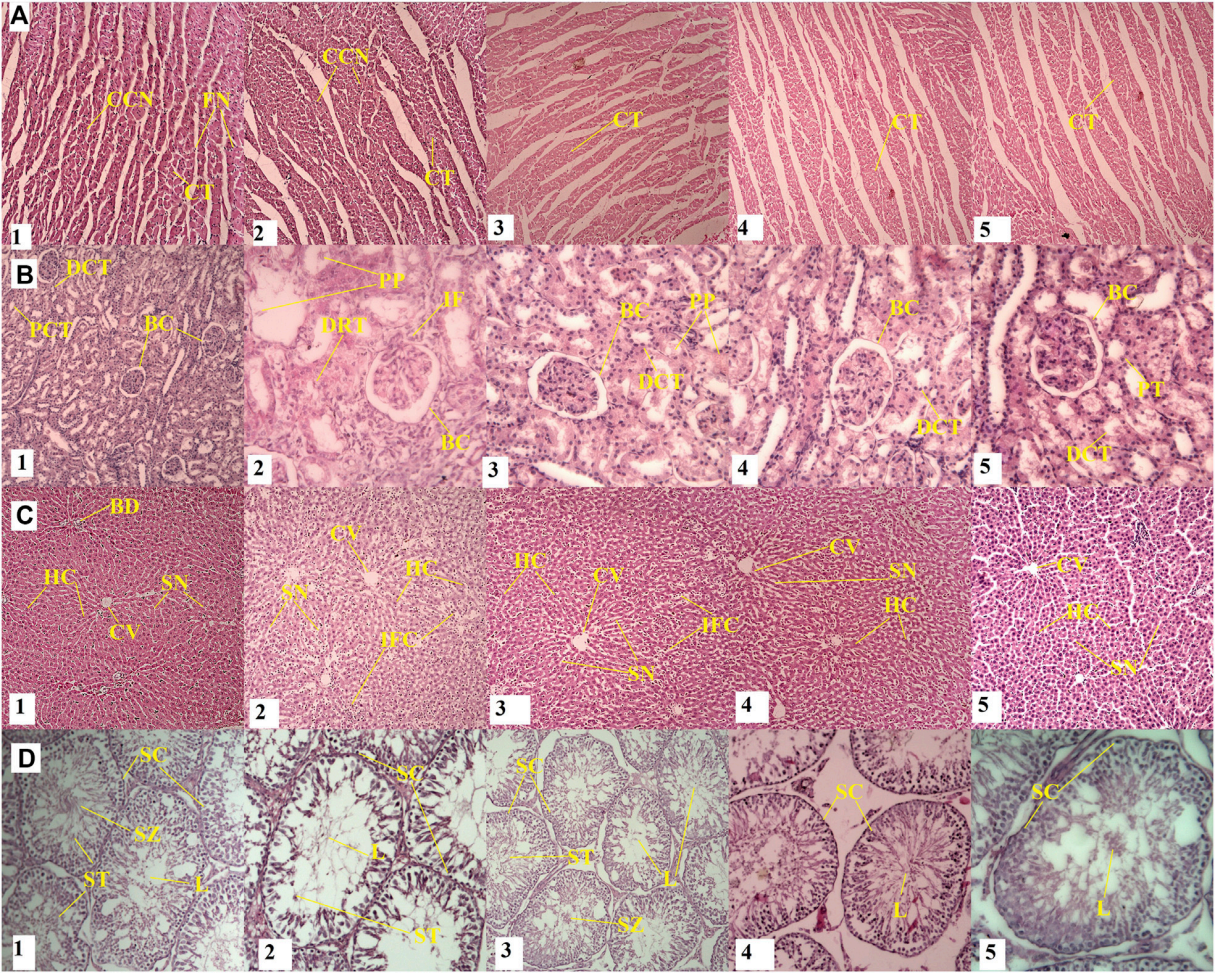
Effect of withametelin on the histology of organs. In the kidneys (B2 and B3), a mild infiltration of mononuclear inflammatory cells around tubules and the degeneration of epithelial cells, renal glomerular capsule space distention, interstitial swelling and congestion, and Pink proteinacious material at 5 mg/kg withametelin (B3) were seen. Tested in the treatment group with withametelin (5 mg/kg) (D2) showed an increase in the interstitial space between the seminiferous tubes, an increase in the lumen size and spermatogenesis stages showed mild sloughing. In the liver, there was an increase in the sinusoidal space, a slight infiltration of inflammatory cells, swelling, and sinusoidal congestion in 5 (C2) and 2.5 (C3) mg/kg groups in comparison to control was observed. No histological changes were seen in heart. A-D represents, heart kidney, liver and testes respectively. 1-5 represents different dose groups i.e., normal or vehicle, 5, 2.5, 1.25, and 0.75 mg/kg. CCN; Cardiac cell nucleus, CT, Cardiac tissue; FN, Fibroblast nucleus; BC, Bowman’s Capsule; DCT, Distal convoluted tubules; PCT, Proximal convoluted tubules; PP, Pink proteinaceous material; DRT, Damaged renal tubule; BD, Biliary duct; HC, Hepatocytes; SN, Sinusoids; CV, Central vein; IFC, Inflammatory cells; SC, Spermatocytes; SZ, Spermatozoa; ST, Spermatids; L, Lumen.

No significant changes were detected in organ weight parameters were observed when comparing control groups and high or low dose groups of both compounds (Table 5). The H&E histological analysis of the withametelin group confirmed the treatment-related changes in liver, kidney and testicle (male) of rats at 5 mg/kg and 2.5 mg/kg when compared to the control group (Figure 4). The toxic effects on the liver and kidneys of males were more profound. In the kidneys, a mild infiltration of mononuclear inflammatory cells around tubules and the degeneration of epithelial cells was observed in 4/5 of males and 2/5 of females. All rats observed renal glomerular capsule space distention. Interstitial swelling and congestion at 5 mg/kg withametelin were also seen. Pink proteinacious material in the lumens of the proximal convoluted tubules were also observed in 3/5 males and 2/5 females. Testes in control groups showed standard morphology of seminiferous tubes with active sperm generation. The treatment group with withametelin (5 mg/kg) showed an increase in the interstitial space between the seminiferous tubes, an increase in the lumen size and spermatogenesis stages showed mild sloughing. Low doses (1.25 mg/kg and 0.75 mg/kg) showed normal seminiferous tubes, the germ epithelium with proliferating germ cells at different stages such as spermatids, spermatocytes, spermatogonia were observed. In the liver, there was an increase in the sinusoidal space, a slight infiltration of inflammatory cells, swelling, and sinusoidal congestion in 5 and 2.5 mg/kg groups in comparison to control was observed. No significant adverse toxicity was observed in other male or female systems of rats.

**TABLE 5:** Relative organ weight values of different organs of withametelin and daturaolone after necropsy.

Parameter	Sex	Relative organ weight per 100 g body weight									
		Withametelin (mg/kg)						Daturaolone (mg/kg)			
		Normal	Vehicle	5	2.5	1.25	0.75	10	5	2.5	1.25
Brain	M	0.45 ± 0.03	0.46 ± 0.02	0.45 ± 0.03	0.47 ± 0.05	0.46 ± 0.02	0.48 ± 0.03	0.46 ± 0.04	0.48 ± 0.04	0.47 ± 0.03	0.47 ± 0.04
	F	0.67 ± 0.04	0.69 ± 0.03	0.66 ± 0.07	0.69 ± 0.07	0.68 ± 0.04	0.69 ± 0.05	0.67 ± 0.06	0.68 ± 0.04	0.67 ± 0.03	0.68 ± 0.03
Kidney	M	0.68 ± 0.02	0.69 ± 0.03	0.68 ± 0.02	0.65 ± 0.02	0.67 ± 0.03	0.70 ± 0.08	0.68 ± 0.04	0.67 ± 0.03	0.68 ± 0.04	0.70 ± 0.07
	F	0.66 ± 0.03	0.66 ± 0.03	0.65 ± 0.03	0.68 ± 0.04	0.67 ± 0.03	0.66 ± 0.04	0.68 ± 0.03	0.66 ± 0.05	0.66 ± 0.05	0.68 ± 0.02
Heart	M	0.27 ± 0.02	0.28 ± 0.04	0.26 ± 0.02	0.29 ± 0.05	0.29 ± 0.04	0.30 ± 0.04	0.27 ± 0.02	0.28 ± 0.02	0.28 ± 0.02	0.29 ± 0.01
	F	0.28 ± 0.02	0.30 ± 0.03	0.27 ± 0.02	0.31 ± 0.08	0.31 ± 0.03	0.30 ± 0.03	0.28 ± 0.01	0.32 ± 0.03	0.32 ± 0.04	0.31 ± 0.02
Liver	M	2.97 ± 0.41	3.23 ± 0.02	3.31 ± 0.41	3.27 ± 0.46	3.33 ± 0.02	3.24 ± 0.02	3.30 ± 0.43	3.20 ± 0.42	3.27 ± 0.05	3.19 ± 0.03
	F	2.73 ± 0.22	3.15 ± 0.06	3.09 ± 0.25	3.23 ± 0.32	3.25 ± 0.08	3.18 ± 0.05	3.12 ± 0.22	3.26 ± 0.31	3.15 ± 0.04	3.23 ± 0.02
Lungs	M	0.33 ± 0.09	0.31 ± 0.05	0.34 ± 0.09	0.32 ± 0.03	0.32 ± 0.07	0.33 ± 0.05	0.35 ± 0.07	0.34 ± 0.02	0.33 ± 0.03	0.35 ± 0.04
	F	0.41 ± 0.02	0.39 ± 0.05	0.42 ± 0.03	0.44 ± 0.05	0.40 ± 0.05	0.38 ± 0.08	0.41 ± 0.04	0.43 ± 0.04	0.41 ± 0.06	0.42 ± 0.07
Spleen	M	0.16 ± 0.01	0.18 ± 0.02	0.16 ± 0.05	0.18 ± 0.05	0.17 ± 0.02	0.16 ± 0.02	0.17 ± 0.06	0.19 ± 0.03	0.18 ± 0.03	0.17 ± 0.03
	F	0.17 ± 0.01	0.17 ± 0.03	0.18 ± 0.04	0.19 ± 0.03	0.17 ± 0.06	0.17 ± 0.04	0.19 ± 0.07	0.18 ± 0.02	0.18 ± 0.05	0.18 ± 0.02
Testis	M	0.98 ± 0.04	0.96 ± 0.02	0.96 ± 0.05	0.99 ± 0.05	0.98 ± 0.04	0.98 ± 0.02	0.96 ± 0.04	0.98 ± 0.06	0.99 ± 0.02	0.95 ± 0.03
Ovary	F	0.83 ± 0.05	0.84 ± 0.04	0.86 ± 0.06	0.84 ± 0.03	0.83 ± 0.06	0.84 ± 0.07	0.85 ± 0.03	0.86 ± 0.04	0.85 ± 0.05	0.85 ± 0.05
Uterus	F	0.26 ± 0.03	0.25 ± 0.05	0.27 ± 0.04	0.28 ± 0.04	0.26 ± 0.06	0.25 ± 0.04	0.28 ± 0.02	0.27 ± 0.05	0.26 ± 0.05	0.27 ± 0.03

Results are expressed as mean ± SD.

*p < 0.05 was considered significantly different as compared with the controls.

**FIGURE 5:**
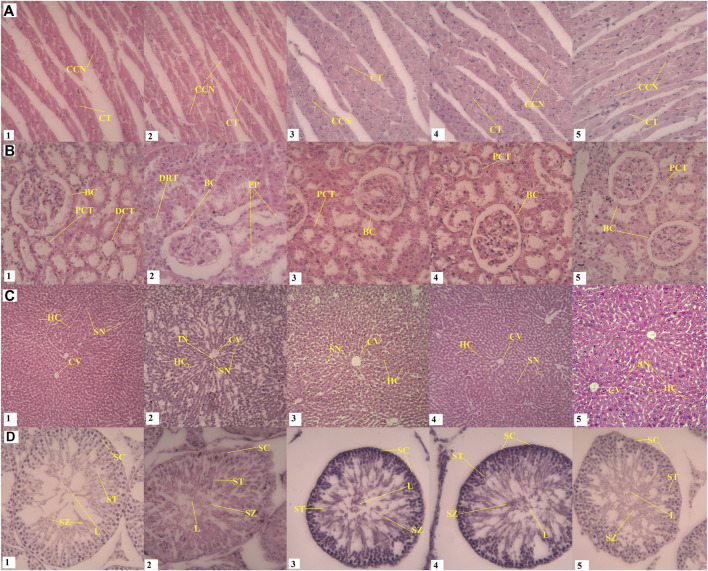
Effect of daturaolone on the histology of organs. Kidney histopathology (B2) showed mild tubular kidney nephrosis, cell proliferation, degenerated epithelial cells, adsorption of pink proteinaceous material in the lumen of compressed proximal tubules. Mild tubular renal necrosis and vacuolar cytoplasm from the tubular lumen base membrane. Testes (D2) showed very few interstitial spaces between the seminiferous tubules and a decrease in the size of the lumen. In D3 and D4, a slight increase compared to normal seminiferous tubes with germ epithelium that contain germ cells were found proliferating at different stages at higher pace were observed which might signify aphrodisiac character. In the liver (C2), increase in sinusoidal space, mild inflammatory cell infiltration, edema and sinusoidal congestion were observed in comparison to control (C1) No histological changes were seen in heart. A-D represents heart, kidney, liver and testes respectively. 1-5 represents different dose groups i.e., normal or vehicle, 10, 5, 2.5 and 1.25 mg/kg. CCN; Cardiac cell nucleus, CT, Cardiac tissue; FN, Fibroblast nucleus; BC, Bowman’s Capsule; DCT, Distal convoluted tubules; PCT, Proximal convoluted tubules; PP, Pink proteinaceous material; DRT, Damaged renal tubule; BD, Biliary duct; HC, Hepatocytes; SN, Sinusoids; CV, Central vein; IFC, Inflammatory cells; SC, Spermatocytes; SZ, Spermatozoa; ST, Spermatids; L, Lumen.

Treatment-related changes in rats liver and kidney and some more pronounced positive effects on testis (male) were also observed by H & E histology analyses in the group of daturaolone treated (10 mg/kg) as compared to the control group. No gender differential toxic affect was seen. In the kidney, the glomerular space of the kidney was distended and the epithelial cells around the tube were degenerated. In rats with high dose (10 mg/kg), the kidneys showed mild tubular kidney nephrosis characterized by increased cell proliferation, degenerated epithelial cells with the adsorption of pink proteinaceous material in the lumen of compressed proximal tubules (Figure 5). In addition, we found a mild tubular renal necrosis, vacuolar cytoplasm and pyknotic nuclei separated from the tubular lumen base membrane. The treated group of daturaolone (10 mg/kg) testes showed very few interstitial spaces between the seminiferous tubules and a decrease in the size of the lumen. At lower doses (1.25 and 0.75 mg/kg), a slight increase compared to normal seminiferous tubes with germ epithelium that containing germ cells were found proliferating at different stages at higher pace were observed which might signify its aphrodisiac character. In the liver, increase in sinusoidal space, mild inflammatory cell infiltration, edema and sinusoidal congestion were observed in 10 mg/kg groups in comparison to control. There were no significant adverse toxic effects observed at high dose on any other systemic organs of rats either male or female. Despite its lipophilic nature, no toxic effects were seen on the brain and tissues with normal neurocytes having well-defined nuclei in brain cortex were observed when compared to control.

## 4 Discussion

It is desirable to determine toxic end points at the beginning of the development of new molecules. Although secondary metabolites are mainly used for their biological benefits, their toxicity may limit their usage. Acute toxicity assessment fixed dose procedure (OECD guideline 420) at 5, 50, and 300 mg/kg with only the sighting study was performed. After years of controversy and discussion, the LD_50_ test was finally abolished at the end of 2002. Three substitute animal tests, the Fixed Dose Procedure, the Acute Toxic Class Method and the Up and Down procedures have been set up to significantly improve the welfare of animals (Botham, 2004). The method of fixed doses used in our study originates from a method proposed by the British Society of Toxicology (1984). As a final point, a reduction in the number of animals used for acute toxic testing and, if possible, avoiding fatalities are the final objective. Instead of using lethality, it is based on a clear toxic signal observed at a certain fixed dose level that is valid at the termination point in the European Economic Community (EEC) acute toxicity classification system (Lipnick et al., 1995).

In acute studies, no treatment related changes by daturaolone at all doses were observed while weight loss, abnormal posture, abnormal gait, diarrhoea and lethargy were observed in 300 mg/kg withametelin group as compared to control group. Changes in weight clearly showed some damage caused by test material (Liu et al., 2016). This weight decline might be due to the reduction in the use of food which was also observed in 28 days repeated dose study, indicating that withametelin might have adverse effects on the reduction of appetite. This was also due to mild diarrhea observed in first few days of a single dose, which was accompanied by lethargy. Withametelin was characterized as GHS category 4 drug with evident toxicity at 300 mg/kg while daturaolone as GHS category 5 or unclassified compound which might show toxicity at 2000 mg/kg in acute toxicity assessment. Weight differences are influenced by several factors such as growth hormone and somatostatin changes in hormone status as seen in sexual steroid secretion which influence maturation patterns and subsequential changes in neurotransmitters affecting food consumption. Furthermore, the rat environment and type of treatment can also be stressful for rat, resulting in weight changes (Zainul Azlan et al., 2020). Changes in weight are considered to be indicators of adverse reactions caused by chemical components. In the subacute toxicity, body weight gain, food and water consumption were normal in the daturaolone groups but significantly reduced in high dose (5 mg/kg withametelin) males and females. Meanwhile, during the treatment period, food consumption in both genders was significantly reduced during the treatment period. Insufficient nutrition may contribute to weight loss. However, a significant reduction in weight in high doses may be partly due to a reduction in food consumption (Li et al., 2022). In repeated dose-toxicity studies, dose-dependent transient salivation was also observed in groups treated with withametelin. The salivation affects two different dopamine receptors i.e., dopamine receptor (D1) and an invertebrate-specific D1-like dopamine receptor (D1L) (Li et al., 2018). D1 receptors act on the epithelial cells of the salivary glands acini for inward fluid transport. D1L modulates each acinus to excrete saliva from acini to the saliva ducts. It is likely that this is due to the action of myoepithelial cells and valves to pump/gate. Withametelin has the ability to dock with dopamine receptors (Baig et al., 2020) but checking the specific interaction with D1 receptors is proposed. Abnormal gait in last week was also seen with piloerection in rats given withametelin (5 and 2.5 mg/kg). Studies on withametelin has been associated with brain in our previous work ameliorating oxidative stress in brain and spinal cord (Khan et al., 2021b; 2021a). High doses of isolated compounds can be toxic due to their potential to react causing prooxidative effects or damaging beneficial ROS concentrations that normally exist under physiological conditions and are necessary for optimal cell function (Bouayed and Bohn, 2010). Aggressive antioxidative stress undermines the angiogenesis of endothelial cells in the brain and the functions of blood brain barrier (Mentor and Fisher, 2017). The automatic gait process is mediated by the brain stem to the spinal cord (Takakusaki, 2017). Daily dosing of 5 mg/kg might have cause detrimental effect on cerebral cortex causing abnormal gait but histopathological analysis suggested otherwise.

The hematopoietic system is one of the most vulnerable organs to the effects of hazardous substances and plays a significant role in both pathological and normal situations. The blood profile gives important details on how the body responds to stress and injury. As a result, a considerable amount of harmful substances are first exposed to blood cells. When findings from animal research are extrapolated (Shakya et al., 2020), changes in the hemotopoietic system have a better prognostic value for human toxicity. Unlike water or vehicle-treated rats, hematopoietic parameters were modified with withametelin and daturaolone treatment, which could prove their dangerous effect on the hematopoietic system. We observed significant decrease in daturaolone (10 mg/kg) and withametelin (5 and 2.5 mg/kg) treated RBC parameters suggesting possible indication of normocytic and microcytic anemia, respectively. A significant increase in platelets in the high dosed groups of withametelin only is contrary to previous findings where withametelin increased coagulation time via single oral administration (Baig et al., 2020). Thus, secondary thrombocytosis is the cause of this rise in platelet count. Both substances caused neutrophilic leukocytosis, as evidenced by an increase in haematological markers and a neutrophil infiltration of the liver by histology neutrophils. There are not many neutrophils that live in the liver, although they frequently patrol the sinusoids of the liver. They are the primary phagocyte types in charge of pathogen clearance and are capable of being quickly recruited into the liver after acute liver damage. However, overly active neutrophils may harm the liver. Neutrophils are therefore seen as a double-edged sword in acute liver inflammation (Tang et al., 2021).

ALT and AST were disturbed significantly by high dose withametelin while only ALT levels were changed by daturaolone which might have resulted in altered permeability of the hepatocellular membrane causing a release of these soluble cytosolic enzymes. Enzymes escape out from the hepatocytes causing elevation in the blood. ALP is considered to be a cholestasis inducer enzyme of hepatobiliary origin used widely to detect abnormal bile flow and has minimal activity in normal liver tissues. The ALP value in rats is reduced by food consumption and body weight reduction or frequent exposure to toxicological doses which might have lead to a decline in value (Ramaiah, 2007). Therefore, it is inconclusive to predict whether lowered ALP levels by withametelin were due to decreased food consumption or cholestasis has occurred. Low hemoglobin levels in daturaolone (10 mg/kg) might be due to abnormally high bilirubin. Bilirubin is generated from the stepwise catalytic degradation of hemoglobin by heme oxygenase and biliverdin reductase (Chung et al., 2019). This strengthens our previous hypothesis of anemia by withametelin. Withametelin and daturaolone also lowered glucose levels. Daturaolone is known to inhibit α-glucosidase (Bawazeer et al., 2020b) while withametelin inhibits both α-amylase and α-glucosidase enzymes (unpublished data). Natural components promote antioxidant defense and suppress the production of proinflammatory cytokines, producing biological activity by improving antioxidant defence mechanisms. Low-cellular stressor dosages of chemicals lead to an adaptive response that increases the antioxidant capacity of cells (Rong et al., 2020).

Hyperthyroidism noted in withametelin (5 and 2.5 mg/kg) groups might be associated with reduction in serum creatinine caused by reduction in overall muscle mass (Iglesias et al., 2017). The decrease in cholesterol and triglycerides observed could be due to increased LDL receptor expression in hepatocytes, increased activity of liver enzymes that reduce lipids, resulting in a reduction in low-density lipoprotein levels by hyperthyroidism. Thyroid hormones also increase expression of A1 apolipoprotein, the main component of high density lipoprotein (Malik and Hodgson, 2002). Histopathological changes in the testicles may also be caused by hyperthyroidism, which delays the development of the Leydig cells and has a negative impact on sperm formation. Other effects secondary to thyrotoxicity such as decrease in total body water and exchangeable potassium, increase in cardiac output and increase in systolic blood pressure needs detailed investigation and validation.

## 5 Conclusion

In summary, GHS classification model characterize withametelin as category 4 and daturaolone as category 5 compound in acute toxicity studies. After the 28-days repeated oral dose subacute toxicity study, NOAEL of withametelin is 1.25 mg/kg and of daturaolone is 5 mg/kg. Weight loss, hyperthyroidism, high platelets, neutrophils, ALT and AST in high dose (5 and 2.5 mg/kg) withametelin groups was prominently observed. Abnormal biochemical changes mainly in ALT levels and glucose indices were seen in high dose (10 mg/kg) daturaolone groups. Sex specific testicular histological changes were also observed by both compounds. Long term studies with more behavioral, biochemical, histopathological and hormonal parameters are proposed to strengthen the findings.

## Data Availability

The raw data supporting the conclusions of this article will be made available by the authors, without undue reservation.
